# Comparison of the efficacy and safety of Transarterial chemoembolization with and without Apatinib for the treatment of BCLC stage C hepatocellular carcinoma

**DOI:** 10.1186/s12885-018-5081-3

**Published:** 2018-11-19

**Authors:** Shiguang Chen, Wenchang Yu, Kongzhi Zhang, Weifu Liu

**Affiliations:** Department of Interventional Oncology, Fujian Cancer Hospital & Fujian Medical University Cancer Hospital, Fuzhou, No. 420 Fuma Road, Fuzhou, 350014 China

## Abstract

**Background:**

Hepatocellular carcinoma (HCC) is a common cancer worldwide, with a poor prognosis. Most patients are diagnosed at advanced stages and are only eligible for palliative therapy. Therefore, this study aimed to evaluate the safety and efficacy of transcatheter arterial chemoembolization (TACE) combined with apatinib (TACE-apatinib) treatment and TACE-alone treatment for Barcelona Clinic Liver Cancer stage C HCC.

**Methods:**

We retrospectively reviewed 80 consecutive patients with BCLC stage C HCC who received TACE-apatinib or TACE-alone as the initial treatment. We compared the clinical and laboratory outcomes, imaging findings at 1 and 3 months after TACE, tumor response, time to progression (TTP), overall survival (OS), and adverse events between both groups.

**Results:**

The overall response rate was higher in the TACE-apatinib group than in the TACE-alone group at 1 and 3 months after treatment (66.7% vs 39.6%, respectively, *P* = 0.020; 45.8% vs 17.6%, respectively, *P* = 0.021). The median TTP and OS in the TACE-apatinib group were longer than those of the TACE-alone group (TTP: 6.3 months vs 3.5 months, respectively, *P* = 0.002; OS: 13.0 months vs 9.9 months, respectively, *P* = 0.041). Apatinib-associated side effects such as hypertension, hand-foot syndrome, oral ulcers, proteinuria, and diarrhea were more prevalent in the TACE-apatinib group than in TACE-alone group (*P* < 0.05).

**Conclusion:**

Compared to TACE-alone treatment, TACE-apatinib increased the TTP, OS, and tumor-response rate at 1 and 3 months after treatment of BCLC stage C HCC without any significant increase in severe adverse events.

## Background

Hepatocellular carcinoma (HCC) is among the 5 most-common cancers worldwide and has a poor prognosis [1]. Only a few patients with HCC are candidates for curative measures such as surgical therapies (liver transplantation and hepatectomy) and percutaneous ablation [2, 3]. In most cases, HCC is diagnosed at an advanced stage, even at Barcelona Clinic Liver Cancer (BCLC) stage C, and such patients are only eligible for palliative therapy. According to the BCLC clinical staging system, the standard treatment for BCLC stage C HCC is oral sorafenib administration [4]. Sorafenib is a polykinase inhibitor with anti-proliferative and anti-angiogenic effects, which can prolong the overall survival of patients with advanced HCC by almost 3 months as compared to the best supportive care; in addition, it has shown good tolerance in patients with HCC [5, 6]. However, its high cost and drug resistance limit its use in advanced liver cancer.

Apatinib is a small-molecule tyrosine kinase inhibitor that has higher selective inhibition of vascular endothelia growth factor (VEGF) receptor-2 than sorafenib, leading to inhibition of VEGF-mediated endothelial cell migration and proliferation, reduction of tumor microvascular density, and inhibition of tumor growth [7]. The phase II clinical trial of apatinib in the treatment of BCLC stage B or C HCC indicated that apatinib was effective in the treatment of intermediate and advanced HCC [8]. In addition, our phase II study demonstrated that apatinib was well tolerated and effective in intermediate and advanced HCC [9]. Therefore, this study aimed to evaluate the safety and efficacy of transcatheter arterial chemoembolization (TACE) combined with apatinib administration (TACE-apatinib) and TACE-alone for the treatment of BCLC stage C HCC.

## Methods

### Patient selection

From March 2016 to October 2017, we retrospectively reviewed 80 consecutive patients with BCLC stage C HCC who underwent TACE-apatinib or TACE-alone as the initial treatment in our institution. Of these, 27 patients underwent TACE-apatinib treatment and 53 underwent TACE-alone treatment. Patients were diagnosed with BCLC stage C HCC on the basis of the criteria of the American Association for the Study of Liver Diseases (AASLD) guidelines [10].On confirmation of BCLC stage C HCC, the patient was informed of sorafenib administration as the recommended treatment. For those who refused to receive sorafenib, TACE-apatinib or TACE alone was recommended as an alternative treatment on the basis of previous phase II studies [8, 9, 11]. The most-common reason for rejection of sorafenib treatment was the high cost, as sorafenib is not on the list of drugs covered by medical insurance of China.

The diagnosis of HCC was established on the basis of suggestions from the AASLD [12] and the European Association for the Study of the Liver [13] by using clinical data, contrast-enhanced computed tomography (CT) or magnetic resonance imaging (MRI) findings, and serum levels of AFP. In 11 patients (13. 8%), the diagnosis was confirmed by ultrasound-guided fine-needle biopsy. The inclusion criteria were as follows: Eastern Cooperative Oncology Group performance score of 0–2, Child-Pugh liver function class A or B, adequate hematologic function (leukocyte count ≥3000/mm^3^, platelet count ≥50,000/mm^3^), and adequate liver function (serum total bilirubin level ≤ 2 mg/dL). The exclusion criteria were as follows: infiltrative or diffused HCC, incomplete course of apatinib (full course lasts for 4 weeks), previous systemic anticancer therapy, and loss to follow-up.

A week prior to treatment, we recorded a comprehensive medical history of all patients, measured their serum AFP levels, and determined their HBsAg status. No hepatitis C virus infection or alcohol addiction was noted in any patient. Abdominal contrast-enhanced CT or MRI and chest CT were included in the initial workup in all patients.

### TACE treatment

Each treatment was performed by an interventional radiologist with at least 5 years of TACE experience. In all patients who underwent TACE, the Seldinger technique was used for hepatic artery catheterization. Using digital subtraction angiography, the catheter or microcatheter was inserted from the right femoral artery and guided to the hepatic artery or its branches for angiography. Subsequently, tumor-feeding arteries were superselected on the basis of our understanding of the tumor blood supply indicated by hepatic arteriography. An emulsion of 3–20 mL Lipiodol (Guerbet Laboratories, Aulnay-Sous-Bois, France) and a chemotherapy agent such as 30 mg epirubicin and150 mg oxaliplatin was injected into the tumor-feeding arteries, which were then embolized using 25–130 mg gelatin sponge particles (GSP) (Ailikang Pharmaceutical Co., Ltd. Hangzhou, China). The GSP was available in three size ranges (150–350 μm, 350–560 μm, and 560–710 μm). The size of GSP depended on the superselected hepatic artery and tumor size, and the most-common size used was 350–560 μm. The aim of chemoembolization was to achieve complete arterial blockage in the arteries supplying the tumor. In some patients with serious portal thrombosis, wide tumor distribution, hepatic arterioportal fistula, or hepatic arterialvenous fistula, the full doses of embolic agents were not administered due to the high risk of failure to recover liver function after treatment.

The TACE treatment was repeated at least 40 days after the first treatment in patients with survival lesions according to the mRECIST [14]. TACE was not repeated until liver failure or tumor progression of the target lesions was observed.

### Apatinib administration

Upon agreeing to participate in the study, all patients in the TACE-apatinib group were orally administered apatinib at an initial dose of 500 mg/day for the first time 2 days after TACE. When the patients encountered grade 3–4 drug-related AEs according to the Common Terminology Criteria for Adverse Events version 4.0, the drug dose was adjusted to 250 mg/day or stopped for several days. After the adverse events were relieved, the patients were recommended to resume daily intake of 500 mg/day apatinib. Treatment continued until patient death, significant disease progression, drug intolerance, or withdrawal of consent from the study.

### Efficacy assessment

Contrast-enhanced CT or MRI was performed 1 and 3 months after treatment, and the results were assessed according to the mRECIST to evaluate tumor response. Two radiologists compared the follow-up images with baseline images to evaluate tumor response to treatment. Based on the results, the patients were categorized into four groups: CR, partial response, stable disease, and progressive disease. The ORR was calculated as the rate of CR plus partial response. TTP was defined as the time between initiations of TACE treatment to the time of disease progression. OS referred to the time from initiation of the first TACE treatment to death or the date of the last follow-up for patients who were alive and censored.

### Statistical analysis

All data analyses were performed using the SPSS software (version 18.0; SPSS, Chicago, IL, USA). Independent sample *t*-test, chi-square test, Kaplan–Meier survival analysis, and log–rank test were used to assess the differences between the two groups. In addition, multivariate Cox-regression was used to analyze overall survival. Values of *P* < 0.05 were considered statistically significant.

## Results

### Patient characteristics

The mean age of patients in the TACE-apatinib group was 45. [8] ± 11.0 years and in the TACE-alone group was 54.4 ± 11.9 years. Although the TACE-apatinib group was younger than the TACE-alone group, there was no significant difference between the two groups. The sex distribution in both groups was as follows: TACE-apatinib group, 23 men and 4 women, and TACE-alone group, 43 men and 7 women. We did not observe any significant difference in the distribution of age, sex, hepatitis B surface antigen (HBsAg) expression, alpha-fetoprotein levels, Child-Pugh class, maximum HCC size, number of HCC foci, extrahepatic metastasis, vascular invasion, and number of TACE procedures between the two groups (Table 1). The date of the last follow-up was May 31, 2018, and the median follow-up period was 12 months. Six patients were lost to follow-up after 6–36 months.

**Table 1:** Baseline characteristics of two groups’ patients

	TACE-apatinib	TACE alone	*P*
Patients, *n*	27	53	
Age(years), mean ± SD	45.8±11.0	54.4 ± 11.9	0.654
Sex, *n* (%)			
Male	23 (85.2)	43 (81.1)	0.453
Female	4 (14.8)	10 (18.9)	
HBsAg expression			
Positive, *n* (%)	26 (96.3)	49 (92.5)	0.659
Negative, *n* (%)	1 (3.7)	4 (7.5)	
Child-Pugh class			
A, *n* (%)	21 (77.8)	48 (90.6)	0.112
B, *n* (%)	6 (22.2)	5 (9.4)	
AFP, ng/ml			
≤ 400, *n* (%)	10 (37.0)	23 (43.4)	0.382
> 400, *n* (%)	17 (63.0)	30 (56.6)	
Maximum HCC size(cm), mean ± SD	12.11 ± 3.98	10.59 ± 4.30	0.419
Number of HCC foci			
1, *n* (%)	3 (11.1)	14 (26.4)	0.137
2, *n* (%)	1 (3.7)	4 (7.5)	
3, *n* (%)	0 (0)	3 (5.7)	
> 3, *n* (%)	23 (85.2)	32 (60.4)	
Extrahepatic metastasis, *n* (%)	9 (39.1)	16 (30.1)	0.483
Vascular invasion, *n* (%)			
The number of TACE, mean ± SD	22 (81.5)	46 (86.8)	0.374
	2.19±1.04	2.34 ± 3.99	0.371

Abbreviations: *SD* standard deviation, *HBsAg* Hepatitis B surface antigen, *AFP* alpha-fetoprotein, *HCC* hepatocellular carcinoma, *TACE* transarterial chemoembolization;

During the follow-up, one patient received secondary surgical treatment in the TACE-apatinib group. A total of 19 patients were treated with additional radiofrequency ablation, including 8 patients in the TACE-alone group and 11 patients in the TACE-apatinib group. A total of 6 patients received additional radiotherapy, including 4 patients in the TACE-alone group and 2 patients in the TACE-apatinib group. There was no significant difference in supplementary therapy between the two groups.

### Tumor response

**Fig. 1:**
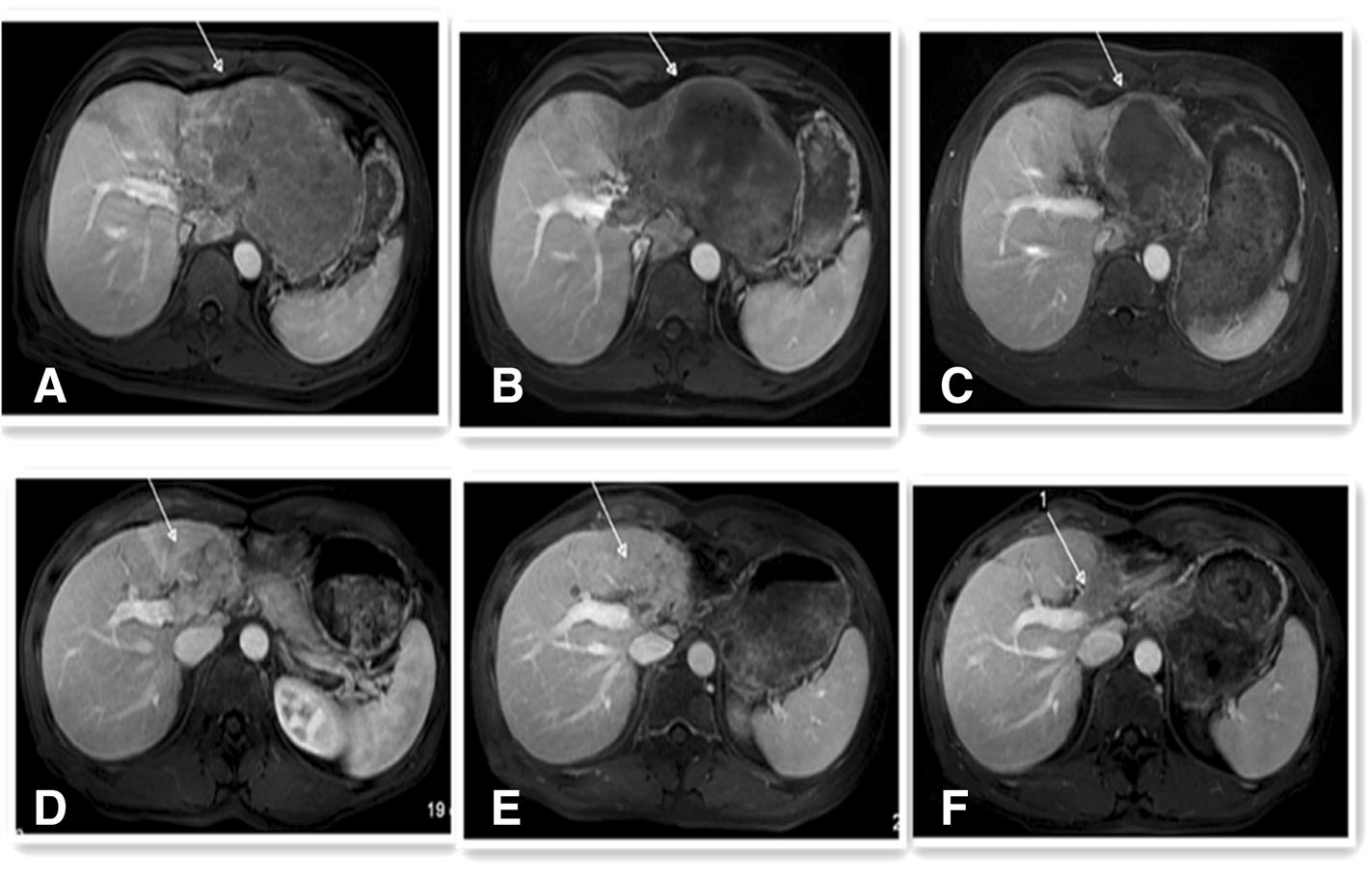
Results of portal vein phase imaging with enhanced abdominal MRI and the changes in tumors in a representative case before TACE and at 1, 3 months after treatment in both two groups. MRI with tumor enhancement pre-TACE in a patient later treated with TACE-apatinib (**a**), and TACE-alone (**d**). Note the enhanced integrity of the tumor in both A and D. MRI with tumor enhancement scan in the patient treated with TACE-apatinib (**b** and **c**) and TACE-alone (**e** and **f**) at 1 and 3 months after treatment. Partial enhancement can be seen in Fig. b, c, e and f, and the strengthening part are obviously smaller than that of Figure a and d

Abdominal MRI scans before TACE and at 1 and 3 months after TACE showed changes in tumors in the two groups (Fig. 1 a–f). We evaluated tumor response in all 80 patients at 1 month after treatment and in 58 patients at 3 months after treatment in both groups using the modified Response Evaluation Criteria in Solid Tumors (mRECIST) (Table 2). Complete response (CR) was not observed in any of the patients. The rate of overall response (ORR) was significantly higher in the TACE-apatinib group than in the TACE-alone group (18/27 [66.7%] vs 21/53 [39.6%], respectively, *P* = 0.020] at 1 month after treatment. The ORR was significantly higher in the TACE-apatinib group than in the TACE-alone group (11/24 [45.8%] vs 6/34 [17.6%], respectively, *P* = 0.021] at 3 months after treatment.

**Table 2:** In the two groups were followed up for 1 and 3 months objective response

	Treatment group	CR	PR	SD	PD	ORR(%)	X^2^	P
1 month	TACE-apatinib	0	18	6	3	18/27 (66.7)	5.236	0.020
	TACE alone	0	21	18	14	21/53 (39.6)		
3 months	TACE-apatinib	0	11	3	10	11/24 (45.8)	5.395	0.021
	TACE alone	0	6	5	23	6/34 (17.6)		

Abbreviations: *CR* complete response, *PR* partial response, *SD* stable disease; *PD* progressive disease, *ORR* objective response rate, *TACE* transarterial chemoembolization

### Time to progression (TTP)

**Fig. 2:**
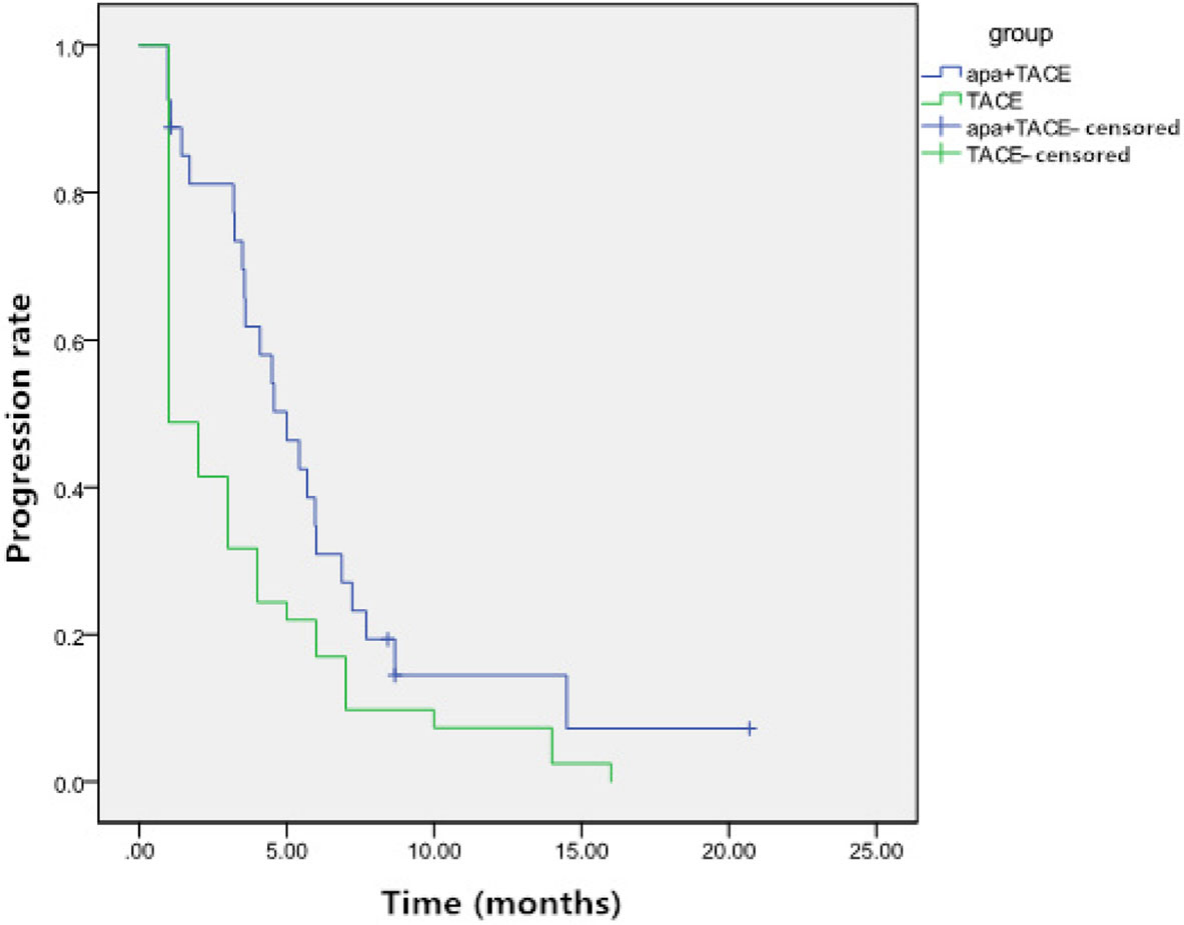
Time to progression (TTP) survival curves of patients with unresectable hepatocellular carcinoma in the two groups. The median TTP in the Apatinib+TACE group was significantly longer than that of the TACE alone group (TTP: 6.3 months vs 3.5 months, *P* = 0.002)

The median TTP was 6.3 months (95% confidence interval [CI]: 4.2–8.4 months) in the TACE-apatinib group and 3.5 months (95% CI:2.3–4.7 months) in the TACE-alone group. The median TTP in the TACE-apatinib group was significantly longer than that in the TACE-alone group (*P* = 0.002, Fig. 2).

### Overall survival (OS)

**Fig. 3:**
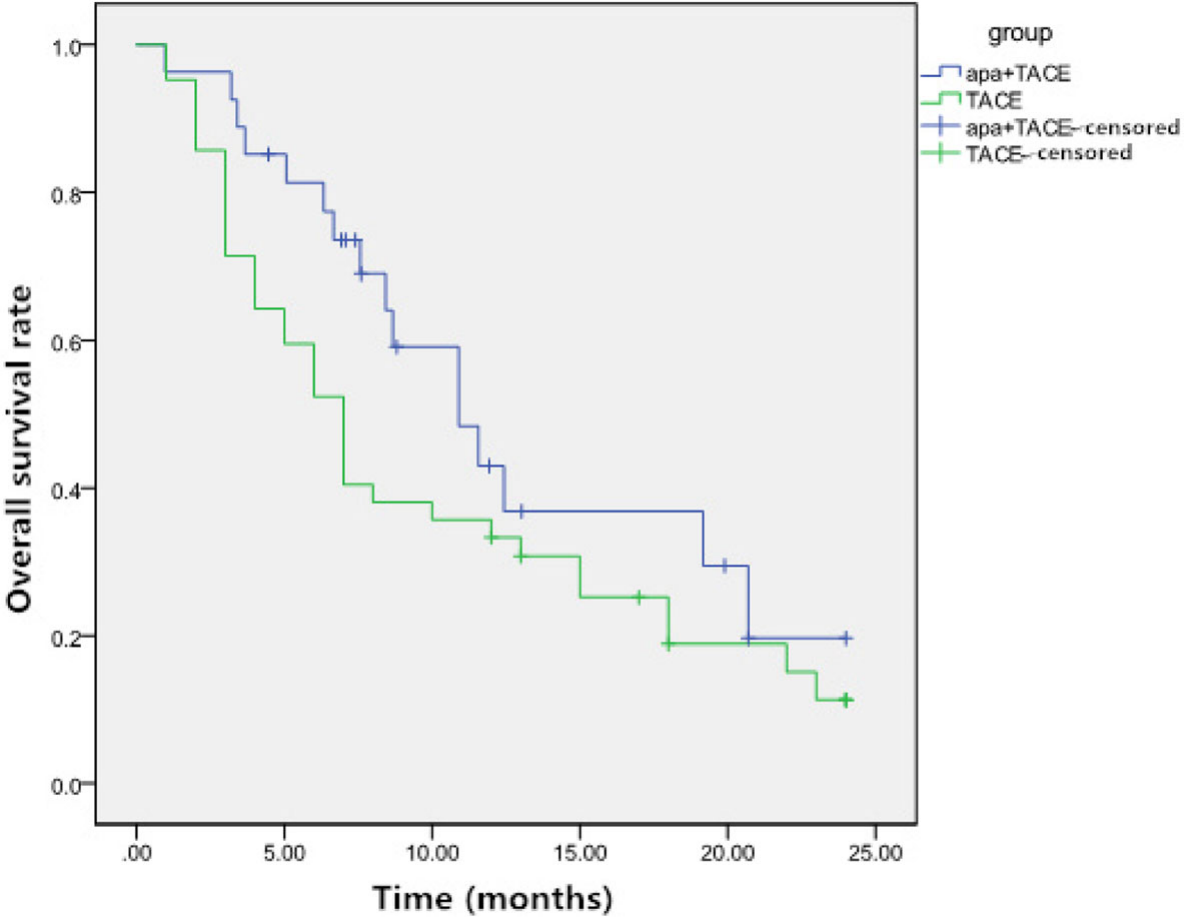
Overall survival(OS) curves of patients with unresectable hepatocellular carcinoma in the two groups. The median survival period in the Apatinib+TACE group was significantly longer than that of the TACE alone group (OS: 13.0 vs 9.9 months, *P* = 0.041)

The median survival period was 13.0 months (95% CI: 9. 8–16.2 months) in the TACE-apatinib group and 9.9 months (95% CI: 7.5–12.3 months) in the TACE-alone group. Survival rates at 1 and 2 years were 43.0 and 19.7%, respectively, in the TACE-apatinib group, and 33.3 and 11.3%, respectively, in the TACE-alone group (*P* = 0.041, Fig. 3). Multivariate Cox regression analyses revealed that overall response at 1 month after treatment was associated with OS. The length of the OS increased with an improvement in the overall response at 1 month after treatment (Table 3).

**Table 3:** Variables independently associated with overall survival of all patients by multivariate analysis

Variables	SE	*P* value	Exp(B)	95% CI for Exp(B)
Overall response	0.304	0.015	2.086	1.151–3.781

### Adverse events (AEs)

AEs associated with treatment in the two groups are listed in Table 4. There were no treatment-related deaths or grade 4 AEs. In the TACE-apatinib group, apatinib-related adverse reactions included hypertension (grade 1 in 6 patients, 22.2%; grade 2 in 13 patients, 48.1%), hand-foot syndrome (grade 1 in 4 patients, 14.8%; grade 2 in 9 patients, 33.3%; and grade 3 in 9 patients, 33.3%), oral ulcers (grade 2 in 1 patients, 3.7%; grade 3 in 4 patients, 14.8%), proteinuria (grade 1 in 2 patients, 7.4%; grade 2 in 3 patients, 11.1%; and grade 3 in 3 patients, 11.1%), and diarrhea (grade 1 in 2 patients, 7.4%; grade 2 in 5 patients, 15,5%); however, these AEs did not occur in the TACE-alone group. The difference in AEs was significant between the two groups (*P* < 0.05). All apatinib-related AEs were manageable by providing symptomatic treatment and/or adjusting the drug dose, which did not affect the treatment. The most-common AEs were hematologic toxicity, post-embolization syndrome (including fever, abdominal pain, nausea, and vomiting), and liver dysfunction in both groups, however, there was no significant difference between the two groups.

**Table 4:** Treatment-related AEs in patients in two groups

AEs	TACE-apatinib, *n* (%)			TACE-alone, *n* (%)			*P* value
	Grade 1	Grade 2	Grade 3	Grade 1	Grade 2	Grade 3	
Leukocytes	2 (7.4)	2 (7.4)	0	4 (7.5)	0	0	0.133
Haemoglobin	0	2 (7.4)	1 (3.7)	0	2 (3.8)	0	0.272
Platelets	2 (7.4)	2 (7.4)	0	4 (7.5)	0	0	0.133
Bilirubin	3 (11.1)	4 (14.8)	0	11 (19.5)	3 (5.7)	0	0.264
ALT	3 (11.1)	5 (18.5)	0	12 (22.6)	7 (13.2)	0	0.402
AST	3 (11.1)	2 (7.4)	0	7 (13.2)	0	0	0.132
Fever	3 (11.1)	11 (40. 7)	0	7 (13.2)	18 (34.0)	0	0.833
Abdominal pain	4 (14.8)	3 (11.1)	0	9 (17.0)	5 (9.4)	1 (1.9)	0.891
Nausea/Vomiting	2 (7.4)	0	0	3 (5. 7)	0	0	1.000
Diarrhoea	2 (7.4)	5 (18.5)	0	2 (3.8)	0	0	0.004
Hypertension	0	6 (22.2)	13 (48.1)	0	0	0	0.000
Hand-foot syndrome	4 (14.8)	9 (33.3)	9 (33.3)	0	0	0	0.000
Oral ulcer	0	1 (3.7)	4 (14.8)	0	0	0	0.000
Proteinuria	2 (7.4)	3 (11.1)	3 (11.1)	0	0	0	0.000

A *P*-value of < 0.05 is considered significant

## Discussion

The BCLC staging system has been widely used in clinical practice and many clinical trials for the treatment of HCC [15]. BCLC Stage C HCC includes vascular invasion and/or extrahepatic metastasis. The AASLD/European Association for the Study of the Liver and the European Organisation for Research and Treatment of Cancer recommend the use of sorafenib for the treatment of both of vascular invasion and/or extrahepatic metastasis in BCLC stage C HCC; however, the Japanese Society of Hepatology recommends the use of hepatic arterial infusion chemotherapy, sorafenib, TACE, and resection for the treatment of vascular invasion and sorafenib for the treatment of extrahepatic metastasis [15–17]. In addition, in China, TACE, systemic therapy (sorafenib or FOLFOX4 chemotherapy), and radiotherapy are recommended for treatment of vascular invasion, whereas systemic therapy (sorafenib or FOLFOX4) and radiotherapy are recommended for the treatment of extrahepatic metastasis [18]. As such, there is no universal standard treatment protocol for BCLC stage C HCC, although sorafenib and TACE are the common treatments.

TACE may stimulate tumor neovascularization by blocking tumor-feeding arteries and causing local hypoxia [19]. In addition, VEGF is known to be the strongest angiogenic factor in HCC patients [20]. After TACE,VEGF expression in tumor tissues around the residual tumor increases and tissue invasion and metastasis are enhanced [21]. These factors form the basis of disease progression or the emergence of new lesions. Therefore, inhibiting the over expression of VEGF in tumor cells induced by TACE is important for improving the long-term effect of TACE. As a multi-kinase inhibitor, sorafenib is currently the only targeted oral drug approved to treat advanced HCC. Apatinib is a novel VEGFR-2 inhibitor that has 10 times the affinity to bind to VEGFR-2 tyrosine kinase as compared to sorafenib [7].

In the current study, comparison of TACE-apatinib and TACE-alone treatments for BCLC stage C HCC showed significant differences in the median TTP, OS, and tumor response at 1 and 3 months after treatment. These differences may be attributed to apatinib, which inhibits the over expression of VEGF in tumor cells induced by embolization after TACE, thereby blocking the migration and proliferation of vascular endothelial cells, decreasing tumor microvessel density, and inhibiting tumor growth. The phase III ORIENTAL clinical trials showed that the median TTP and OS was 2.8 and 6.5 months, respectively, and the ORR was 3.3% in the sorafenib-treated patients with advanced HCC in the Asia-Pacific region [6]. In the current study, the median TTP and OS of patients with advanced HCC treated with TACE-apatinib were 6.3 and 13.0 months, respectively, and the ORR was 66.7%. Although these are not head-to-head comparative study, the results still suggest that clinicians tend to use TACE-apatinib treatment for advanced HCC as an alternative to sorafenib.

In our study, multivariate Cox regression analyses revealed that overall responses at 1 month after TACE treatment were associated with OS. This finding is consistent with that of Kim et al. [22] who analyzed the association between tumor response and survival times of 493 patients with HCC. Patients with BCLC stage C HCC showed vascular invasion and/or extrahepatic dissemination, and most patients in the current study showed vascular invasion. However, multivariate analysis did not indicate vascular invasion and extrahepatic metastasis as independent risk factors for OS, which could be because the limited sample size of this study did not reflect any statistical difference and the possible bias in the statistical results due to the short follow-up time.

No serious complications were observed in any patient in the current study. The side effects associated with apatinib, such as hypertension, hand-foot syndrome, oral ulcers, proteinuria, and diarrhea, were significantly higher in the TACE-apatinib group than in the TACE-alone group (*P* < 0.05); however, all patients tolerated drug therapy well with dose adjustment and symptomatic treatment. There was no significant difference in the side effects associated with TACE between the two groups. In general, the side effects of TACE-apatinib treatment are tolerable.

Despite our important findings, our study had a few limitations that should be considered when interpreting our results. First, the number of enrolled patients was small, especially in TACE-apatinib group, and the follow-up period was only 2 years. Second, there may have been a selection bias owing to the retrospective, single-center nature of the study.

## Conclusions

Our randomized control study showed that TACE-apatinib treatment effectively prolonged TTP and OS, and increased ORR in patients with BCLC stage C HCC. Therefore, prospective randomized controlled study with a larger number of patients in multi-center need to be conducted to prove the effects and safety of TACE combined apatinib in treatment BCLC stage C HCC.

## Abbreviations

BCLC: Barcelona Clinic Liver Cancer
CR: Complete response
CT: Computed tomography
GSP: Gelatin sponge particles
HCC: Hepatocellular carcinoma
mRECIST: modified Response Evaluation Criteria in Solid Tumors
MRI: Magnetic resonance imaging
ORR: the rate of overall response
OS: Overall survival
TACE: Transcatheter arterial chemoembolization
TTP: Time to progression
VEGF: Vascular endothelia growth factor
